# Nuclear receptor agonist-driven modification of inflammation and amyloid pathology enhances and sustains cognitive improvements in a mouse model of Alzheimer’s disease

**DOI:** 10.1186/s12974-018-1091-y

**Published:** 2018-02-15

**Authors:** Brad T. Casali, Erin G. Reed-Geaghan, Gary E. Landreth

**Affiliations:** 1 Department of Neurosciences, Case Western Reserve University School of Medicine, Cleveland, OH 44106 USA; 2 Stark Neurosciences Research Institute, Indiana University School of Medicine, Indianapolis, IN 46202 USA

**Keywords:** Nuclear receptors, RXR, Microgliosis, ABCA1, APOE, Bexarotene, AD

## Abstract

**Background:**

Alzheimer’s disease (AD) is a highly prevalent neurodegenerative disorder characterized by pathological hallmarks of beta-amyloid plaque deposits, tau pathology, inflammation, and cognitive decline. Treatment remains a clinical obstacle due to lack of effective therapeutics. Agonists targeting nuclear receptors, such as bexarotene, reversed cognitive deficits regardless of treatment duration and age in murine models of AD. While bexarotene demonstrated marked efficacy in decreasing plaque levels following short-term treatment, prolonged treatment did not modulate plaque burden. This suggested that plaques might reform in mice treated chronically with bexarotene and that cessation of bexarotene treatment before plaques reform might alter amyloid pathology, inflammation, and cognition in AD mice.

**Methods:**

We utilized one-year-old APP/PS1 mice that were divided into two groups. We treated one group of mice for 2 weeks with bexarotene. The other group of mice was treated for 2 weeks with bexarotene followed by withdrawal of drug treatment for an additional 2 weeks. Cognition was evaluated using the novel-object recognition test either at the end of bexarotene treatment or the end of the withdrawal period. We then analyzed amyloid pathology and microgliosis at the conclusion of the study in both groups.

**Results:**

Bexarotene treatment enhanced cognition in APP/PS1 mice similar to previous findings. Strikingly, we observed sustained cognitive improvements in mice in which bexarotene treatment was discontinued for 2 weeks. We observed a sustained reduction in microgliosis and plaque burden following drug withdrawal exclusively in the hippocampus.

**Conclusions:**

Our findings demonstrate that bexarotene selectively modifies aspects of neuroinflammation in a region-specific manner to reverse hippocampal-dependent cognitive deficits in AD mice and may provide insight to inform future studies with nuclear receptor agonists.

## Background

Alzheimer’s disease (AD) is a prominent neurodegenerative disorder that occurs in later life. Its pathological hallmarks include neurofibrillary tangles composed of intracellular hyperphosphorylated tau and extracellular deposits of β-amyloid (Aβ) species which comprise plaques in the brain parenchyma. Specifically, age-related deficits in Aβ clearance are associated with the accumulation of neurotoxic Aβ species and plaques that are linked to cognitive deficits [1, 2]. Additional pathological consequences that accompany Aβ accumulation include the dysregulated production of pro-inflammatory cytokines from resident microglia and astrocytes [3, 4]. Thus, strategies which enhance the clearance of Aβ, resolution of inflammation, and improve cognition may be of therapeutic utility.

Nuclear receptors are ligand-activated transcription factors that act to coordinate and control the expression of many genes involved in clearance of Aβ and inflammation [5, 6]. ApoE is the major apolipoprotein of the brain and functions to transport cholesterol and phospholipids throughout the brain as high-density lipoproteins (ApoE-HDLs) and plays a prominent role in Aβ metabolism and deposition [7–9]. Formation of ApoE-containing high-density lipoproteins (HDL) is facilitated by lipid transporter ABCA1 which transfers cholesterol and phospholipids to ApoE apolipoproteins. Importantly, the production of ApoE and ABCA1 is transcriptionally controlled by activation of nuclear receptors. The nuclear receptors liver X (LXR) or peroxisome proliferator-activated (PPAR) receptors form obligate heterodimers with retinoid X Receptor (RXR), and upon ligand binding, promote target gene transcription. Clearance of soluble Aβ species is dependent on ApoE and its lipidation state [7]. Importantly, activation of nuclear receptors also promotes transrepression of genes associated with inflammation [10]. Nuclear receptor agonists ameliorate amyloid pathology by decreasing soluble Aβ levels, promoting the phagocytosis of plaques, suppressing the expression of inflammatory genes, and enhancing cognition in various mouse models of AD [11–13].

RXR agonists represent attractive therapeutic agents since RXR acts as the common heterodimer of nuclear receptors LXR and PPAR, and the ligation of RXR promotes transcription of genes associated with Aβ clearance and abrogation of inflammation in AD mouse models [8, 14–16]. Acute treatment with the RXR agonist bexarotene promotes plaque clearance, but chronic bexarotene treatment paradoxically does not affect plaque burden [8]. We thus hypothesized that plaques may reform after their initial clearance by bexarotene. Furthermore, bexarotene may modulate Aβ dynamics since a single dose diminishes soluble Aβ levels by approximately 25% for up to 72 h [8]. The effects of amyloid reformation on pathology and cognition after removal of a nuclear receptor agonist have not been explored in an AD mouse model.

We report here that acute treatment with bexarotene augments production of highly-lipidated ApoE-HDL particles, curtails microglial reactivity, reduces plaque burden, and improves cognition in APP/PS1 mice. Surprisingly, despite cessation of bexarotene treatment, APP/PS1-treated mice continued to demonstrate sustained improvements in cognition and abrogation of amyloid pathology for as long as 2 weeks. Our findings imply nuclear receptor agonist treatment and withdrawal profoundly alter the dynamics of amyloid pathology and cognition in the brain. These findings suggest that intermittent dosing strategies may be possible to mitigate the peripheral side effects of bexarotene.

## Methods

### Reagents

Antibodies were purchased from the following sources and used at the indicated dilutions for Western blots and immunostaining: Iba1 (Wako, #019-19741); 6E10 (Biolegend, #803001); ABCA1 (Novus NB400-105, 1:2500); ApoE (sc-6384; 1:2000), β-actin (sc-1615; 1:10,000), and GAPDH (sc-32233; 1:10,000, all purchased from Santa Cruz Biotechnology); and β-tubulin (Licor #926-42211; 1:10,000). For Aβ ELISAs, Aβ 1-40 HRP-conjugated and Aβ 1-42 HRP-conjugated antibodies were both purchased from BioLegend (# 805407 and #805507, respectively) and both used at 1:5000 dilutions. The anti-Aβ antibody (6E10) was also purchased from BioLegend (#803017) and used to capture Aβ at 1:1000 dilution for ELISAs. The clinical formulation of bexarotene (Targretin™) is a micronized form of the drug which exhibits sustained plasma levels following oral administration and was obtained from (Valeant Pharmaceuticals, 75 mg capsules). Thioflavin S was purchased from Sigma (#T1892).

### Study approval

All animal experiments utilized protocols which were approved by the Case Western Reserve University School of Medicine’s Institutional Animal Care and Use Committee (IACUC).

### In vivo animal treatment study design

**Fig. 1:**
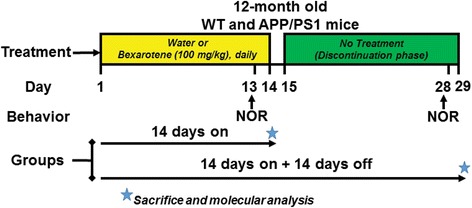
Bexarotene treatment and discontinuation paradigm. Twelve-month-old APP/PS1 or wildtype (WT) littermate mice were divided into two groups: “14 days on” or “14 days on + 14 days off.” Both groups were treated daily for 14 days with bexarotene or vehicle (water), and novel object recognition (NOR) was performed on the 13th day, but the 14 days on the group was sacrificed on the 14th treatment day. The 14 days on + 14 days off group was then subject to a discontinuation phase from bexarotene treatment for days 15 to 29, with NOR performed on day 28 and sacrifice on day 29. Stars indicate molecular endpoints for each group consisting of protein, ELISA, and IHC analyses. Each treatment phase was performed separately, and groups were combined for behavioral and molecular analyses

Transgenic APPswe/PS1Δe9 (APP/PS1) mice, which carry APPswe K595N/M596L mutation and a deletion of exon 9 of human presenilin 1 [17], were purchased from Jackson Laboratories (MMRRC stock #34832) and bred in-house to generate experimental animal cohorts. Animals were housed in a facility under 12-h light and dark cycle and had access to food and water ad libitum. Male and female transgenic (Tg) or wildtype (WT) mice and littermate controls aged 12 months were used in all studies. For bexarotene treatment and its discontinuation (see Fig. 1), mice were divided into two groups: 14 days bexarotene treatment alone (14 days on) or 14 days on bexarotene treatment and 14 days off bexarotene (14 days on + + 14 days off). Targretin capsules were dispersed in water and administered at 100 mg/kg by oral gavage daily for 14 days for both groups, and water alone served as vehicle control. After 14 days of either vehicle or bexarotene treatment, the 14 days on + 14 days off group was discontinued from vehicle or bexarotene treatment without any handling for an additional 14 days. Novel object recognition (NOR) was performed on either day 13 of the treatment for both groups or on day 27 for the 14 days on + 14 days off group only. Mice in both groups were euthanized on either day 14 or day 29 for according to IACUC guidelines. For behavioral testing, we used mixed sex groups for each time point. For “14 days on” time point, the number and sexes of mice used were WT vehicle (*n* = 10, six males and four females), Tg vehicle (*n* = 4, all males), and Tg bexarotene (*n* = 6, four males and two females). For “14 days on + 14 days off,” the number and sexes of mice used were WT vehicle (*n* = 14, seven males and seven females), Tg vehicle (*n* = 9, eight males and one female), and Tg bexarotene (*n* = 10, six males and four females). For molecular analyses, seven or more animals were sampled for each treatment group. For 6E10 and Iba1, the following sample sizes and sexes were analyzed: 14 days on (vehicle, *n* = 5 M/2F; bexarotene, *n* = 5 M/4F) and 14 days on + 14 days off (vehicle, *n* = 6 M/1F; bexarotene, *n* = 4 M/3F).

### Novel object recognition behavioral test

NOR was assessed and scored as previously described [15]. Briefly, on either the 13th day of bexarotene treatment or on the 27th day of bexarotene discontinuation, mice were placed in a chamber with two identical objects placed opposite to each other in the boxes’ corners. Mice were allowed to explore the chamber freely for 10 min, and the time spent investigating objects (*T*) was recorded. Three hours later, one object was replaced with a different object, but of similar size and shape, and mice were returned to the chamber and allowed to explore the objects for 5 min, and the duration of *T* spent investigating was recorded. Between tests, each chamber and object was cleaned to eliminate residual odor. Following treatment discontinuation, mice were subsequently retested with different objects than those used previously. Animals not performing the test (i.e., not interacting with any objects) were excluded from the analysis (*n* = 1). Novel object preference is displayed as the relative discrimination index via the formula: [(*T*_novel_ − *T*_familiar_)/(*T*_total_)].

### Collection of tissue

Approximately 3 to 6 h following final bexarotene administration or on the final day of the discontinuation phase, animals were euthanized. The brains were removed and divided into two hemispheres, and one hemisphere was used for immunostaining, and the other hemisphere was used for molecular analysis of protein, RNA, or Aβ levels. For molecular analysis, the midbrain and cerebellum were discarded and the remaining tissue (cortex and hippocampus) was snap-frozen on dry ice and stored at − 80 °C until analysis.

### Immunostaining

One hemisphere of the brain was drop fixed in 4% paraformaldehyde overnight at 4 °C. The brain was then cryoprotected in increasing PBS/sucrose gradients, embedded, and stored at − 80 °C until sectioning. The brains were then sectioned on a cryostat (Leica) at 40 μm as freely floating sections in cryoprotection buffer (30% glycerol [*v*/*v*] and 30% ethylene glycol [*v/v*] in PBS) and stored at − 20 °C until staining. Thioflavin-S staining was performed as previously described [8]. For 6E10 and Iba1 staining, citric acid (Sigma, 251275) was used for antigen unmasking with modifications for free-floating sections but essentially performed as previously described [8]. For quantification of all immunostaining, two matched medial and lateral sections from each animal were used, images were captured on an inverted microscope with similar exposure and gain settings for all samples, and images were quantified with ImageJ (NIH) in a blinded manner. Thioflavin-S hippocampal plaque counts were normalized to total imaged hippocampal area determined using Adobe Photoshop.

### Extraction of Aβ and Aβ ELISA

Aβ species from the brain homogenates were isolated as previously described [18]. In brief, 0.4% diethylamine (DEA) was added to homogenized tissue, and soluble proteins were collected from the supernatant following ultra-speed centrifugation. The remaining pellet was sonicated in cold formic acid (FA), and FA-solubilized proteins were collected from the supernatant following another round of ultra-speed centrifugation. Soluble (DEA) or insoluble (FA) extracts were then subjected to Aβ ELISAs with 6E10 as capture antibody (see the “Reagents” section, above). Detection of either Aβ 1-40 or Aβ 1-42 was used with specific detection antibodies conjugated to HRP. ELISA levels were normalized to total protein in each extract (either soluble or insoluble), and results represented as fold change relative to vehicle-treated animals.

### Western blotting and native gel electrophoresis

For cell protein analysis, cultured cells were frozen at − 80 °C until analysis, whereupon whole cell lysis was performed with ice-cold RIPA buffer (50 mM Tris-HCl pH 7.6, 150 mM NaCl, 1% Triton X-100 [*v/v*], 1% sodium deoxycholate [*v/v*], 0.1% SDS [*v/v*], and 2 mM EDTA) supplemented with protease inhibitor cocktail (Sigma, #P8340). The brain tissue was homogenized with a handheld homogenizer in cold tissue homogenization buffer (2 mM Tris pH 7.4, 250 mM sucrose, 0.5 mM EDTA, and 0.2 mM EGTA) with fresh protease inhibitors added at the time of homogenization. Protein levels were evaluated by resolving brain homogenates or astrocyte lysates on 4–12% Bis-Tris gels (Life Technologies), with samples reduced. To analyze lipidated ApoE-HDL particles, the brain homogenates were resolved on 4–12% Tris-Glycine gels (Life Technologies) under non-denaturing conditions. All gels were transferred to PVDF membranes and blocked, and primary antibodies were incubated overnight at 4 °C (see the “Reagents” section above for dilutions). Proteins were visualized using secondary HRP-conjugated antibodies, and immunoblots were analyzed using ImageJ (NIH).

### RNA isolation and quantitative, real-time RT-PCR

To extract total RNA, an aliquot of brain homogenate was mixed with RNABee (Amsbio) reagent, and the solution was incubated on ice with chloroform. The resultant aqueous layer was removed, and RNA was isolated using PureLink RNA Mini Columns (Life Technologies) according to manufacturer’s protocol. Contaminating genomic DNA was removed, and complementary DNA (cDNA) was reverse-transcribed with High Capacity RNA to cDNA conversion kit (Life Technologies) from equal concentrations of RNA across samples. Pre-amplification (14 cycles) was subsequently performed using TaqMan PreAmp Master Mix (Applied Biosystems) with desired targets. Pre-amplified, diluted cDNA was then used to run quantitative, real-time RT-PCR on a StepOne Plus Real-Time PCR System with desired TaqMan probes (all from Applied Biosystems; probe numbers are available upon request). Expression levels of mRNA were determined relative to housekeeping genes GAPDH and 18S RNA, and data were normalized to a wildtype calibrator sample. Statistics were performed at the relative (delta *C*_t_) level [19].

### Primary astrocyte isolation and in vitro treatment

Mixed glial cultures were obtained from neonatal mouse pups aged P0 to P3 from both sexes as previously described [11]. In brief, the brains were removed (with meninges intact) and minced, then the tissue was incubated in trypsin/EDTA to dissociate cells. Cells were resuspended in DMEM/F12 media supplemented with 10% heat-inactivated fetal bovine serum (FBS) and incubated at 37 °C under 5% CO_2_ for 2 to 3 weeks. Following incubation, astrocyte monolayers were enzymatically detached with trypsin. Astrocytes were seeded into 6-well plates in 10% DMEM/F12 media, allowed to adhere for approximately 15 min, and media was aspirated, and new serum-containing media was replaced to eliminate residual microglia. For all cell treatments, astrocytes were serum starved for at least 24 h in DMEM/F12 media. To model bexarotene discontinuation in vitro, astrocytes were treated with 10 nM bexarotene or DMSO. Following 24-h treatment, one set of DMSO and bexarotene-treated astrocytes were frozen at − 80 °C (bexarotene treatment). The media from a parallel set of DMSO and bexarotene-treated astrocytes were removed, the cells were washed twice with PBS, and serum-free DMEM/F12 was replaced, and cells returned to the incubator. Following 24 h, media was removed, and the final set of DMSO and bexarotene-treated astrocytes were frozen at − 80 °C (post-bexarotene treatment). Cells were then lysed, and protein expression was analyzed via Western blot detailed above. For quantification, Abca1 and ApoE protein levels were quantified via ImageJ relative to loading control. Values for each group were then normalized to vehicle-treated samples and represented as fold change.

### Statistics

All error bars represent the standard error of the mean (SEM), unless otherwise stated. Where appropriate, a one- or two-way Student’s *t* test was used and noted in the figure legends. Statistics were determined using GraphPad Prism 5. *P*-values were considered statistically significant when they were less than 0.05.

## Results

### Study treatment design

To ascertain the longevity of the effects of RXR agonist bexarotene on amyloid pathology and cognition in APP/PS1 transgenic mice, we designed a treatment paradigm consisting of two treatment groups (Fig. 1). Twelve-month-old transgenic APP/PS1 mice and their wildtype (WT) littermates were divided into two groups: “14 days on” or “14 days on + 14 days off.” Both groups were treated daily with bexarotene (or vehicle control, water) for 14 days; however, the “14 days on” group was sacrificed on the 14th day of bexarotene treatment while the “14 days on + 14 days off” group entered a discontinuation phase where mice received no bexarotene or vehicle treatment for 14 additional days. This second group was sacrificed on day 29. Both groups were assessed by novel object recognition (NOR) tests on days 13 and 28.

### Bexarotene significantly enhances and maintains short-term memory improvements in vivo

**Fig. 2:**
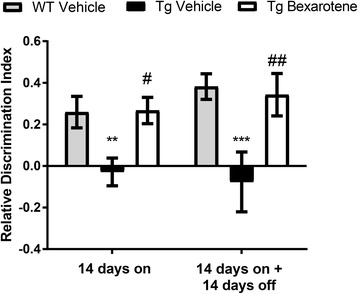
Bexarotene treatment corrects short-term memory deficits, and cognitive improvements are maintained after removal in vivo. Novel object recognition behavioral assay is displayed as the relative discrimination index for wildtype (WT)-vehicle and APP/PS1 (Tg)-vehicle or bexarotene-treated mice for each treatment group. The relative discrimination index (see the “Methods” section) was determined as the ratio between the difference in time spent investigating novel and familiar objects to the total time investigating both objects. Relative discrimination indexes of 0 represent lack of discrimination between novel and familiar object while values greater than 0 represent recognition of the novel object. **p < 0.01 and ***p < 0.001 with respect to WT vehicle and #*p* < 0.05 and ##*p* < 0.01 with respect to Tg vehicle, one-way ANOVA with Tukey post hoc test for multiple corrections. Fourteen days on (*n* = 10, 4, 6) and 14 days on + 14 days off (*n* = 14, 9, 10) for WT vehicle, Tg vehicle, Tg bexarotene, respectively. The 14 days on group contains mice that were subsequently tested in the 14 days on + 14 days off group

We wished to test whether bexarotene treatment and its discontinuation influenced short-term memory in a mouse model of AD. We utilized the novel object recognition (NOR) assay, testing the mice days 13 and 28 of the treatment paradigm (Fig. 1). At both time points, we observed significant short-term memory deficits in recognition of the novel object in transgenic vehicle-treated APP/PS1 (Tg) mice compared to their WT vehicle-treated littermates (Fig. 2). No significant differences were observed in WT vehicle-treated mice at either time point. Bexarotene treatment for 14 days resulted in a significant improvement in recognition of the novel object compared to vehicle-treated transgenic animals (Fig. 2, 14 days on, Tg-vehicle vs Tg-bexarotene), consistent with previous findings [20]. Remarkably, APP/PS1 mice discontinued from bexarotene for 2 weeks still exhibited enhanced novel object recognition. These data demonstrate that bexarotene promotes long-lasting improvements in short-term memory deficits in APP/PS1 mice, demonstrable 2 weeks after cessation of bexarotene treatment.

### Bexarotene suppresses microglial reactivity and selectively reduces plaque burden in the hippocampus

**Fig. 3:**
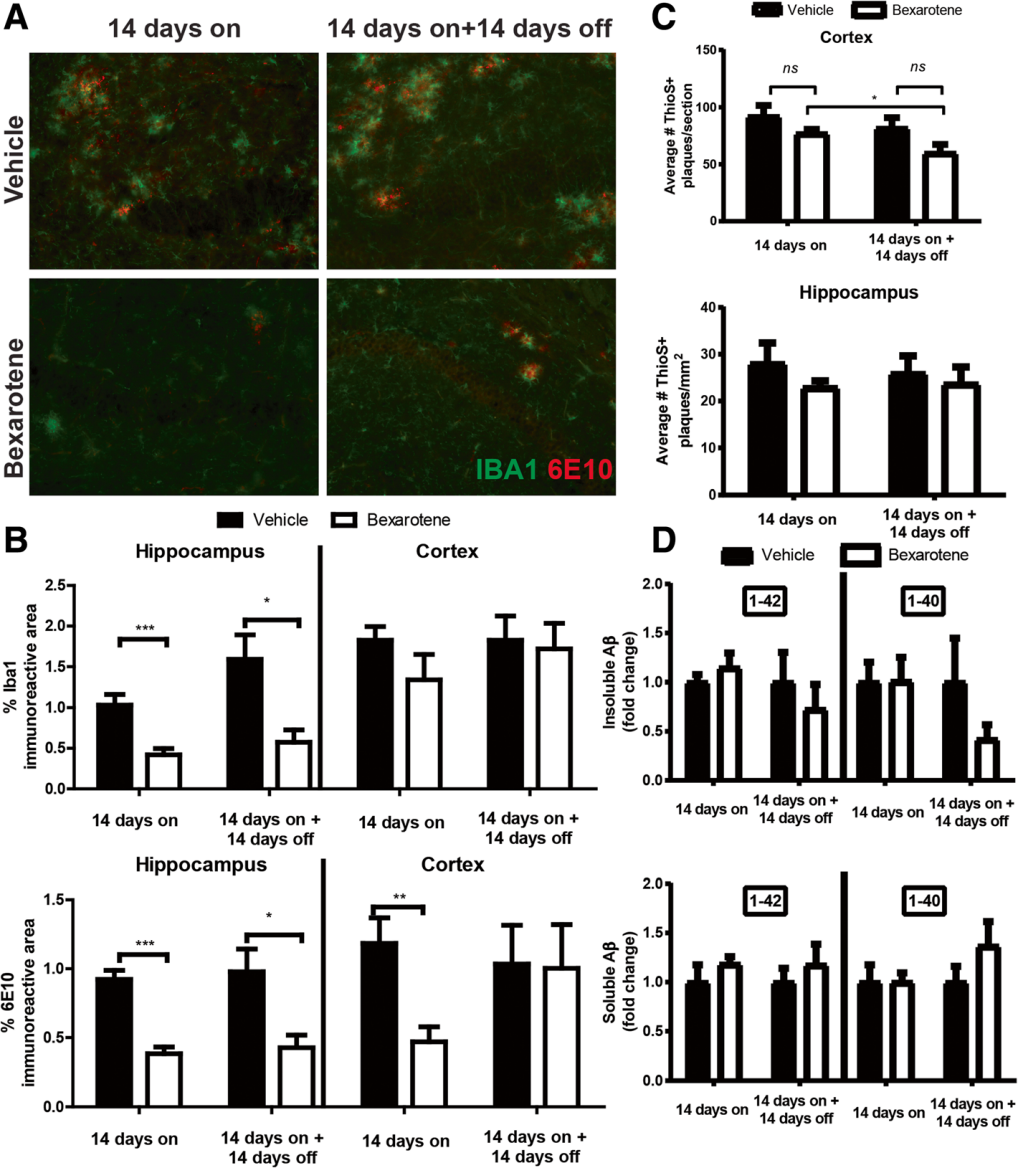
Bexarotene selectively reduces microgliosis and decreases plaque burden in the hippocampus. **a** Representative hippocampal images stained with Iba1 (green) and 6E10 (red) antibodies from vehicle- or bexarotene-treated animals from each treatment group. **b** Quantification of the hippocampal (left) and cortical (right) Iba1 or 6E10 immunoreactivity. **c** Quantification of either the cortical (top) or hippocampal (bottom) Thioflavin-S-positive (ThioS+) dense-core plaques. **d** ELISA results of Aβ isoforms (1-40 or 1-42) from insoluble (FA, left) or soluble (DEA, right) extracts from the hippocampal and cortical homogenates. *p < 0.05, **p < 0.01, and ***p < 0.001, Student’s *t* test between groups indicated by brackets. ns = not significant. *N* = 7 or more animals/group. Image magnification, × 20

We have demonstrated that bexarotene can influence AD pathology by suppressing the expression of genes associated with inflammation in brain-resident microglia in AD mice [16, 21]. To determine if improved cognition was a result of reduced microglial activation, we performed immunostaining for microglial marker Iba1. Indeed, we observed significant reductions in Iba1 immunoreactivity in bexarotene-treated mice in both “14 days on” and “14 days on + 14 days off” groups; however, these observations were regional-specific and restricted to the hippocampus (Fig. 3a, b, upper graphs). Additionally, co-staining with 6E10, which labels both dense and diffuse plaques, revealed significant reductions in plaque burden in the hippocampus in bexarotene-treated animals in both groups (Fig. 3a, b, lower graphs). While bexarotene significantly reduced 6E10 plaque burden in the cortex in the “14 days on” group, this diminution was temporary as 6E10 plaque burden rebounded to vehicle-treated levels in the “14 days on + 14 days off” group (Fig. 3b, lower graph).

**Fig. 4:**
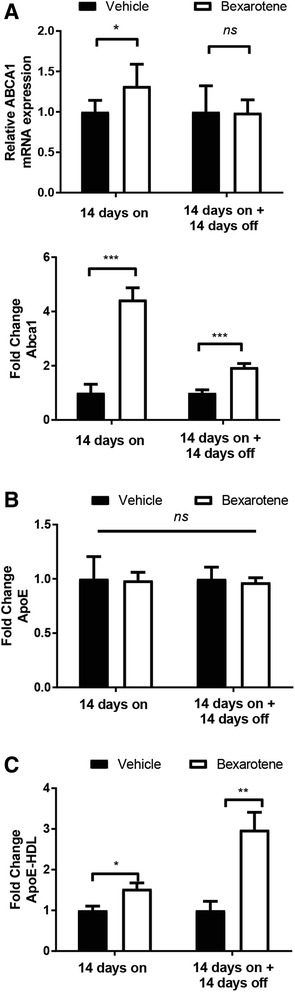
Bexarotene treatment augments Abca1 expression and ApoE-HDL levels in vivo. **a** Relative mRNA expression (top) or protein expression (bottom) of Abca1 in each treatment group from the cortical and hippocampal homogenates. **b**, **c** Quantification of ApoE protein levels (**b**) or lipidated ApoE-HDL particles (**c**) from indicated groups. Results are displayed as fold change vehicle-treated animals from each treatment group. *p < 0.05, **p < 0.01, and ***p < 0.001, Student’s *t* test between groups indicated by brackets. ns = not significant. Error bars for mRNA expression in **a** represent 95% confidence intervals. *N* = 7 or more animals/group

Bexarotene treatment alone, or after its discontinuation, did not significantly modify other metrics of amyloid pathology. Dense-core plaque numbers, represented by Thioflavin-S (ThioS+) staining, were unchanged in both groups following bexarotene treatment in either the brain region (Fig. 3c). We nonetheless observed a significant decrease in cortical ThioS+ plaques between the “14 days on” and the “14 days on + 14 days off” bexarotene-treated groups (Fig. 3c). In a similar fashion, Aβ forms associated with either the insoluble (FA) or soluble (DEA) extracts were unchanged with bexarotene (Fig. 3d). However, this finding could be a consequence of combined cortical and hippocampal tissue homogenization—which may preclude our ability to detect regional-specific effects of bexarotene treatment on specific Aβ levels. Despite modest impacts on amyloid pathology, bexarotene did indeed engage the LXR target gene *Abca1*, resulting in elevated mRNA and protein levels in the brain (Fig. 4a, top and bottom). Protein levels of Abca1 remained significantly upregulated even after bexarotene removal (Fig. 4a, bottom) along with concomitant elevations in lipidated ApoE-HDL levels at both time points (Fig. 4c). Overall ApoE protein levels, however, remained unchanged (Fig. 4b).

Thus, bexarotene acts to suppress microglial reactivity and reduce 6E10 plaque burden in the hippocampus in both treatment groups, an effect which may underlie bexarotene’s salutary impact on cognition that we observed in the NOR test (Fig. 2).

### Protein levels of Abca1 remain significantly elevated following removal of bexarotene in vitro

**Fig. 5:**
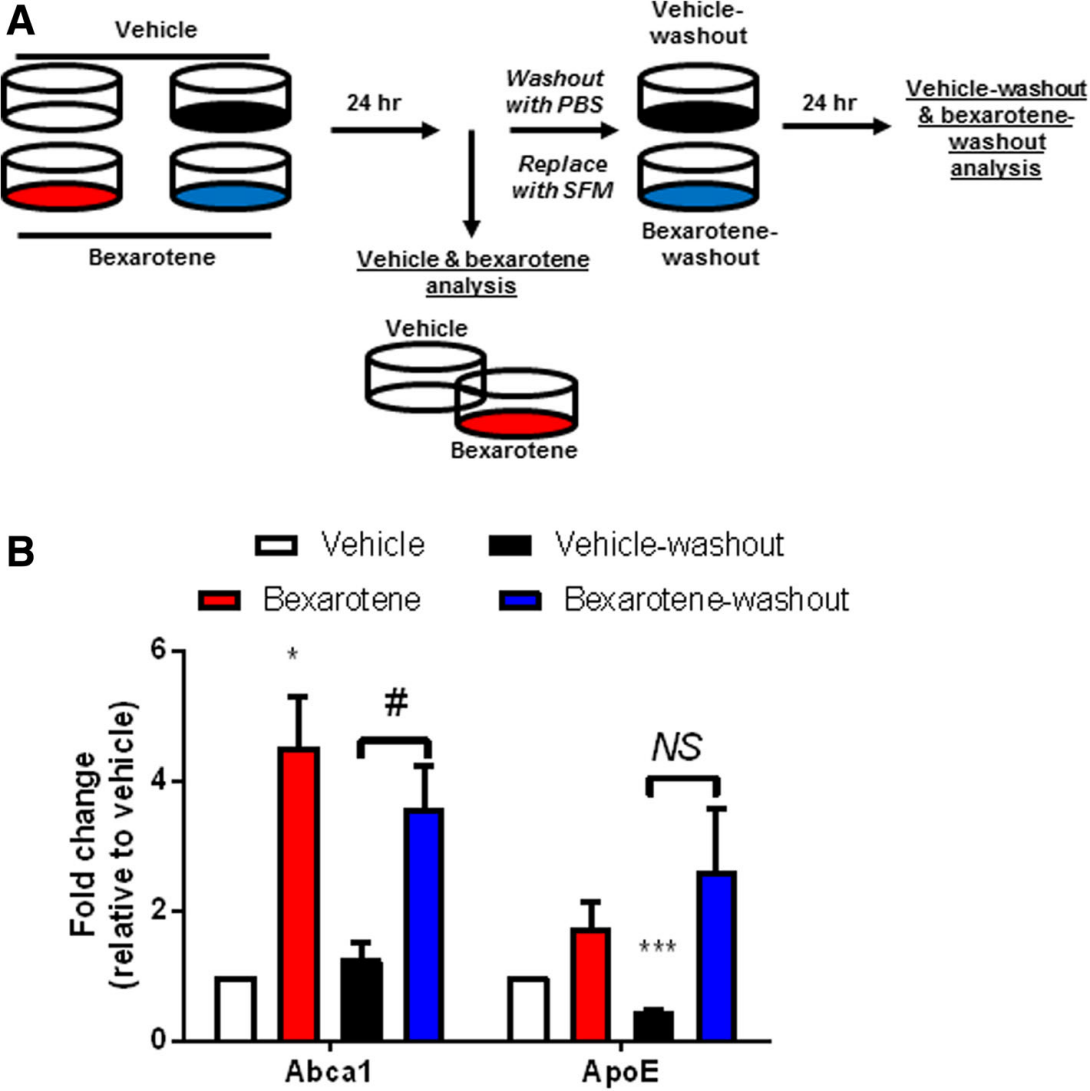
Abca1 remains significantly elevated while ApoE expression does not change after bexarotene removal in vitro. **a** Diagram representing the experimental procedure for analysis of bexarotene removal in primary astrocytes. Four groups of astrocytes were seeded: vehicle (white), bexarotene (red), vehicle-washout (black), and bexarotene-washout (blue). Vehicle (DMSO) or 10 nM bexarotene was applied to astrocytes in serum-free media (SFM) for 24 h after which vehicle and bexarotene samples were then used for protein analysis. Vehicle-washout and bexarotene-washout plates were then washed with PBS, serum-free media replaced, and incubated for an additional 24 h. Following this time point, vehicle-washout and bexarotene-washout samples were then used for protein analysis. **b** Quantification of each protein is represented as fold change relative to vehicle-treated cells. *p < 0.05 and ***p < 0.001, one sample *t* test with respect to vehicle-treated cells. Brackets indicate Student’s two-sample *t* test between indicated samples; #*p* < 0.05 and NS = not significant (exact *p* value = 0.111, Student’s *t* test with Welch’s correction for unequal variances). Data are representative of four separate experiments

Two weeks after bexarotene treatment, we observed significant elevations in the brain Abca1 protein expression in APP/PS1 mice, suggesting that enhanced expression of LXR target genes can be sustained despite the removal of LXR agonist (Fig. 4). To ascertain whether effect could be recapitulated in vitro, we treated primary astrocytes using a paradigm which modeled bexarotene removal and monitored the expression of LXR target genes, *ApoE* and *Abca1*. As shown in Fig. 5a, following isolation of primary astrocytes, we treated two sets of astrocytes with vehicle (DMSO) or bexarotene (10 nM). We performed protein analysis of Abca1 and ApoE 24 h later in one set of astrocytes (this set of experiments is referred to as vehicle and bexarotene analysis) while we washed and replaced the medium in the second set of astrocytes (“vehicle-washout” and “bexarotene-washout” analysis) for an additional 24 h. Protein expression of Abca1 and ApoE was then evaluated in the post-vehicle and post-bexarotene set of astrocytes.

While bexarotene did not induce a significant increase in ApoE protein expression, we did find a significant induction of ABCA1 protein in bexarotene-treated astrocytes after 24 h (Fig. 5b). Following an additional 24 h after vehicle- or bexarotene-washout, Abca1 protein expression in “vehicle-washout” remained unchanged relative to vehicle levels. However, “bexarotene-washout” Abca1 protein expression continued to remain significantly elevated compared to “vehicle-washout” Abca1 levels (Fig. 5b). Interestingly, protein expression of “vehicle-washout” ApoE significantly decreased relative to vehicle levels. “Bexarotene-washout” protein levels of ApoE remained comparable to bexarotene-treated astrocytes, but “vehicle-washout” ApoE levels did not significantly increase compared to “bexarotene-washout” levels (Fig. 5b). Thus, utilizing an in vitro paradigm with primary astrocytes, we demonstrate that Abca1 protein remains significantly elevated while ApoE levels remain unchanged after bexarotene removal—data which support our in vivo results (Fig. 4).

## Discussion

We investigated the dynamics of amyloid pathology and cognition after RXR agonist discontinuation in a mouse model of AD. Specifically, we demonstrate that acute (14-day) bexarotene treatment restores short-term memory deficits of aged APP/PS1 mice and that this 14-day treatment is sufficient to sustain short-term memory improvement even after cessation of drug administration for as long as 2 weeks.

Ample evidence demonstrates the salutary cognitive effects of nuclear receptor agonists in AD mouse models which have been linked to their transcriptional control of genes involved in the clearance of Aβ [7, 22–24] and reviewed in Skerrett et al.[6]. Indeed, deletion of the LXR target gene, Abca1, resulted in an enhanced β-amyloid deposition in AD mouse models and worsened cognition. Conversely, drugs that enhance *Abca1* levels promote clearance of Aβ through elevations in lipidated ApoE-HDL particles and enhance cognition [25]. Abca1 acts to transfer cholesterol and phospholipids to ApoE-based HDL particles. Additionally, HDLs play pivotal roles in cognition in the context of neurodegenerative disorders [26]. Notably, we observed sustained protein expression of the LXR target gene Abca1 and elevated lipidated ApoE-HDL particles (Fig. 4) that mirrored hippocampal-specific reductions in 6E10 plaque burden at both time points (Fig. 3). While Abca1 protein remained elevated in animals discontinued from bexarotene treatment, *Abca1* mRNA levels from these same animals diminished to vehicle-treated levels. This finding could be due to bexarotene’s ability to promote transcription of other LXR target genes that influence the stability of Abca1 at the protein level [27]. Our current data provide direct support for previous observations that bexarotene requires Abca1 expression to ameliorate behavioral impairments and influence hippocampal Aβ levels, involving the generation of ApoE-HDLs in the brain [15], although other mechanisms are also likely to be in play [21].

Nuclear receptor agonists counteract Aβ-induced inflammation molecularly through transrepression of pro-inflammatory gene transcription in microglia [28]. In the current study, we observed that bexarotene blunted the hippocampal microglial activation at both time points (Fig. 3), and this was correlated with improvements in cognition (Fig. 2). Collectively, these data buttress past work implicating salutary effects of nuclear receptor agonists in murine models of AD. Indeed, we have shown previously that bexarotene alone, or in combination with other nuclear receptor agonists, can inhibit the expression of pro-inflammatory genes and improve cognition in various mouse models of AD [13–15, 21].

Secondary to their ability to block pro-inflammatory gene production, nuclear receptor agonists may impact inflammation through production of HDL particles [26, 29]. The anti-inflammatory actions of ApoE-based HDLs are well documented [26, 29, 30]. However, the mechanisms that subserve the effects of ApoE-HDLs on inflammation in the brain are unclear. Abca1 may play an indirect role as APP/PS1 mice lacking Abca1 exhibit enhanced inflammatory genes and, notably, augmented Iba1 reactivity in the hippocampus compared to APP/PS1 mice [15].

With the current “14 days on” bexarotene treatment, we did not observe the drastic reductions in amyloid pathology described in our initial bexarotene study [8], likely due to the analysis of older (1-year-old) mice. It is noteworthy that older mice (approximately 11-month-old) did not display as pronounced differences in amyloid pathology as younger mice (6-month-old) treated with bexarotene [8]. We observed that cortical plaque burden quickly rebounded after bexarotene removal (Fig. 3) which supports our initial findings that bexarotene’s ability to clear plaque diminishes with chronic treatment [8]. Nevertheless, 14 days of bexarotene treatment enhanced short-term memory in the novel object recognition assay in APP/PS1 mice (Fig. 2). Fitz et al. reported that bexarotene also reversed cognitive deficits in novel object recognition in APP/PS1 mice. Though these authors [20] used APP/PS1 mice that also expressed human APOE3/4 isoforms, bexarotene treatment nevertheless restored novel object recognition memory in both groups of mice. Our findings compliment those of Fitz and colleagues and support the targeting of nuclear receptors as a therapeutic strategy [20].

Our initial study emphasized that cognitive improvements may be uncoupled from plaque burden changes and that microglia could play a critical role in bexarotene’s ability to improve cognition in rodents [8, 31]. The current data bolster our initial claims and suggest that microglia reactivity may subserve bexarotene’s benefits in aged AD mice.

While other aspects of amyloid pathology were unchanged with the current model and treatment paradigm, bexarotene unexpectedly demonstrated a marked selectivity in tempering microglial reactivity and reducing plaque burden in the hippocampus. These modifications in AD pathology support the enhanced cognition in both bexarotene-treated and discontinued animals since the hippocampus plays critical roles in object recognition memory in mice [32]. We and others [8, 20] have demonstrated that bexarotene decreases interstitial fluid (ISF) levels of Aβ from the brain and that enhanced clearance of ISF Aβ is facilitated by bexarotene. It is possible that bexarotene alters clearance of ISF Aβ levels to facilitate behavioral improvement over the extended period but was not investigated in the current study. Future studies should be aimed at discerning whether bexarotene withdrawal can sustain cognitive improvement in other hippocampal-dependent behavioral tests.

We and others have previously reported brain region-specific effects of nuclear receptor agonists on amyloid pathology [9, 15, 22]. Although the basis of the region-selective targeting of bexarotene, or other nuclear receptor agonists actions, remains unexplained, other factors may directly or indirectly contribute to these findings. Bexarotene’s ability to reduce Aβ in the hippocampus, but not the cortex, of AD mice was correlated with each brain region’s relative Aβ burden [9]. Additionally, others have reported similar hippocampal-directed effects with LXR agonists in AD mouse models [22]. Moreover, microglial immune-related gene expression varies in a regional-specific manner which can shape the brain’s response to aging-related neurodegeneration [33]. Thus, bexarotene’s selective impact on AD pathology in the hippocampus may be due to the susceptibility of certain brain regions to accumulate Aβ deposits.

## Conclusion

Our findings may inform the design of future studies involving nuclear receptor agonists and also highlight the therapeutic utility of RXR agonists. Recently, bexarotene lowered the brain amyloid in non-ApoE4 carriers in a small, proof-of-concept study in patients with AD, but significant elevations in serum triglycerides were also observed in bexarotene-treated patients [34]. Previously, we demonstrated that elevations in triglycerides due to bexarotene could be counteracted with omega-3 fatty acids in rodents [14]. However, the present data illustrate that bexarotene’s salutary effects on cognition last for up to 2 weeks. This finding raises the possibility that treatment schedules involving the withdrawal of RXR agonists as an additional treatment strategy could be beneficial in a translational environment. This will be particularly critical where significant concerns of cardiovascular perturbations due to bexarotene may outweigh clinical benefits in an elderly population.

## Abbreviations

ABCA1: ATP-binding cassette adaptor 1
AD: Alzheimer’s disease
APOE-(HDL): Apolipoprotein-E (high-density lipoprotein)
Aβ: Amyloid-beta
IBA1: Ionized calcium-binding adaptor molecule 1
ISF: Interstitial fluid
LXR: Liver X receptor
PPAR: Peroxisome proliferator-activated receptor
RXR: Retinoid X receptor
